# A fast methodology for generating skeletal FEM with detailed human geometric features based on CPD and RBF algorithms

**DOI:** 10.1038/s41598-023-35374-3

**Published:** 2023-05-31

**Authors:** Qiuqi Yuan, Binhui Jiang, Xiaoming Zhu, Jingzhou Hu, Yulong Wang, Clifford C. Chou, Shiwei Xu

**Affiliations:** 1 State Key Laboratory of Advanced Design and Manufacturing for Vehicle Body, Hunan University, Changsha, 410082 People’s Republic of China; 2 Shanghai Motor Vehicle Inspection Certification and Tech Innovation Center Co., Ltd., Shanghai, 201805 People’s Republic of China; 3 Department of Oral and Maxillofacial-Head and Neck Oncology, Shanghai Ninth People’s Hospital, Shanghai Jiao Tong University School of Medicine, Shanghai, 200023 People’s Republic of China; 4 Auto Engineering Research Institute, Guangzhou Automobile Group Co., Ltd., Guangzhou, 511434 People’s Republic of China; 5 Bioengineering Center, Wayne State University, Detroit, MI 48201 USA

**Keywords:** Computational biology and bioinformatics, Engineering

## Abstract

Due to the significant effects of the human anatomical characteristics on the injury mechanism of passenger in traffic accidents, it is necessary to develop human body FEM (Finite Element Model) with detailed anatomical characteristics. However, traditional development of a human body FEM is an extremely complicated process. In particular, the meshing of human body is a huge and time-consuming project. In this paper, a new fast methodology based on CPD (Coherent Point Drift) and RBF (Radial Basis Function) was proposed to achieve the rapid developing the FEM of human bone with detailed anatomical characteristics. In this methodology, the mesh morphing technology based the RBF was used to generate FEM mesh in the geometry extracted from the target CT (Computed Tomography) data. In order to further improve the accuracy and speed of mesh morphing, the target geometric feature points required in the mesh morphing process were realized via the rapid and automatic generation based on the point-cloud registration technology of the CPD algorithm. Finally, this new methodology was used to generate a 3-year-old ribcage FEM consisting of a total of 27,728 elements with mesh size 3–5 mm based on the THUMS (Total Human Model for Safety) adult model. In the entire process of generating this new ribcage model, it only took about 2.7 s. The average error between the new FEM and target geometries was only about 2.7 mm. This indicated that the new FEM well described the detailed anatomical characteristics of target geometry, thus importantly revealing that the mesh quality of the new FEM was basically similar to that of source FEM.

## Introduction

Passenger injury caused by traffic accidents is a serious public health issue worldwide^1,2^. Injury mechanism of passenger resulting from different traffic accidents plays an important role in the theoretical basis for solving such public health issue. It has been demonstrated that the human anatomical characteristics have significantly effects on the injury mechanism of passenger^3^. For example, Ridella et al.^4^ reported that obese elderly and child passengers were more likely to be injured than those with normal body characteristics. Therefore, it is greatly significant to study the influences of human anatomical characteristics on injury mechanisms towards the protection of special passengers.

As of today, computational simulation has become one of the main methods to study injury mechanism and establish injury tolerances^5^. In particular, the detailed biomechanical responses of human tissue pertaining to injury severity and location, such as strain and stress, can be predicted by the human body FEM (finite element model). In addition, the human body FEM can accurately characterize the anatomical features of human body. The human body FEM has become one of the most widely used human injury assessment tool in the field of vehicle safety^6^. Therefore, numerous human body FEMs, including the H (Human) model, FHBM (Ford Human Body Model), THUMS (Total Human Model for Safety), GHBMC (Global Human Body Model Consortium) et al., have been developed^7–10^. The process of development for a human body FEM is very complicated and usually includes: 1. creation of human body geometric model from CT (Computed tomography) and MRI (Magnetic resonance imaging) data, 2. FE meshing of human body geometric model, and 3. boundaries, loading, and verification of model. One of the most time-consuming is the meshing process of the geometric model. Existing meshing methods mainly include Delaunay, Advancing Front Technique, Mapping, Sweeping^11,12^. In practical use, the geometric model is decomposed and the meshes are generated mainly through manual interaction. However, for the human body structure with very complex geometric details, the mesh generated by the above methods is of poor quality and can hardly meet the analysis requirements. It requires experienced researchers to improve mesh quality by Laplacian smoothing, elements topology optimization and other operations^13,14^.

Due to the complexity of human body FEM development, the method to obtain new FEM based on the existing basic model through mesh deformation technology has been widely developed. The scaling method was firstly proposed. For example, Vavalle et al.^15^ and Schoell et al.^16^ scaled the 50th male FEM in the GHBMC to obtain a 95th adult male and a 65-year-old male FEM, respectively. In the scaling method, the new FEM is usually obtained by scaling the body parts of the existing basic FEM with different ratios without need of the detailed geometric data of the target geometry. This advantage makes the scaling method widely used in literatures^17–19^. However, the disadvantage of the scaling method is also obvious in aspect that the detailed geometric differences between the body parts of the target FEM and the existing basic FEM are not reflected. Considering this disadvantage of the scaling method, the UMTRI (University of Michigan Transportation Research Institute) and Hunan University recently proposed a mesh morphing method based on feature points and RBF (Radial Basis Functions)^20–23^. Mesh morphing is the smooth transition of a FEM into another similar FEM, where the first model is called the source FEM and the second is called the target FEM. Mesh morphing usually consists of three steps: Firstly, a large number of corresponding feature points are selected at appropriate locations of source FEM and target geometry. Then, the corresponding relationship between them is established through feature points, such as the corresponding relationship between vertices, edges and surfaces. Finally, the meshes of source FEM are mapped to the target geometry by RBF, so the target FEM is obtained. The target FEM generated by mesh morphing can retain the detailed geometric features of target geometry well. However, the limitation of mesh morphing is that a large number of feature points need to be selected manually, which is very time-consuming and laborious. For example, using mesh morphing to generate a ribcage FEM with 27,728 elements usually needs to manually select more than 1,000 feature points^24^. Moreover, once the sequence and number of feature points on the source FEM and the target geometry are inconsistent, the process of mesh morphing cannot be carried out. Considering all of these is necessary to improve such a time-consuming, labor-intensive, and error-prone step.

In this paper, in order to avoid the disadvantage of the mesh morphing method, an automatic generating feature points method using the CPD (Coherent Point Drift) algorithm was proposed. This new methodology was then applied to automatically generate feature points for different human bones, such as ribcage, pelvis, humerus, radius, tibia, and ulna. Furthermore, using the generated feature points, the FE meshing of these human bones was generated by the mesh morphing with RBF. In these applications, generating about 200 and 2000 feature points only takes about 2 s and 24 s, respectively. The quality of FE mesh obtained by using the automatically generating feature points is basically the same as that of FE mesh prior to the morphing. Results reveal that this method capable of generating the feature points automatically is faster and more accurate than the manual extraction.

## Method

The Ethical Committee of Shanghai Ninth People's Hospital approved this retrospective study. And written informed consent was obtained from all the participants. All methods were performed in accordance with relevant named guidelines and regulations.

**Figure 1:**
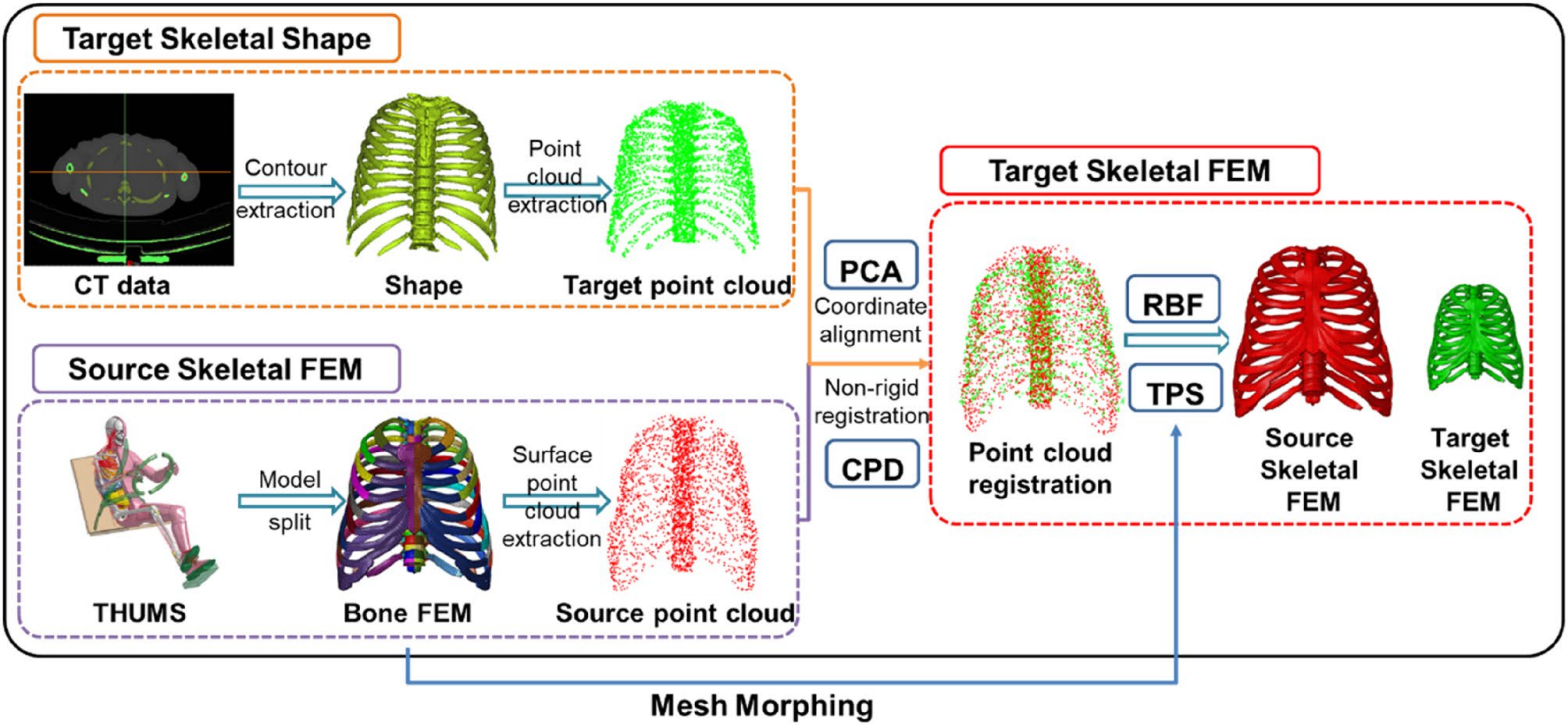
Overview of the fast mesh morphing method.

A whole process from CT data to the FEM using the fast mesh morphing method is shown in Fig. 1. First of all, the geometry of target new model described by point-cloud was extracted from CT data (ribcage, pelvis, humerus, radius, tibia and ulna). In this process, the ribcage was taken as an example of the target new model. Second, the corresponding source FEM of ribcage was split from THUMS, and outermost mesh nodes were extracted as the source point-cloud. Third, a rough registration between the target and source point-clouds was conducted through PCA (Principal Component Analysis) with CPD algorithm to obtain feature points. Finally, the feature points were used to morph the source FEM for target FEM.

### The geometry of target new model

**Figure 2:**
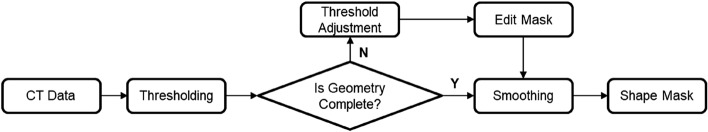
Flow chart of preprocessing part of CT data.

In this study, a CT data of the whole-body for a 3-year-old male child was used to develop the target shape. By adjusting the CT value, the data related to the bone was extracted. Then, the data of bone was thresholding, and smoothed. Sometimes, the CT image quality is not good enough to capture all the geometrical details. For this, threshold adjustment was used to improve the completeness of the geometry. The target shape was then repaired by editing mask. Generally speaking, the complete usable shape mask can be extracted through the preprocessing process described above. The pre-processing process is shown in Fig. 2. The data processed above were exported in the form of a point-cloud.

### The source FEM

The skeletal FEMs were split from THUMS and marked as $FE{M}_{S}$ for source FEMs. Then the outermost nodes of each $FE{M}_{S}$ were extracted as the source point-cloud for each bone and marked as ${P}_{S}$.

### Automatically generating feature points

**Figure 3:**
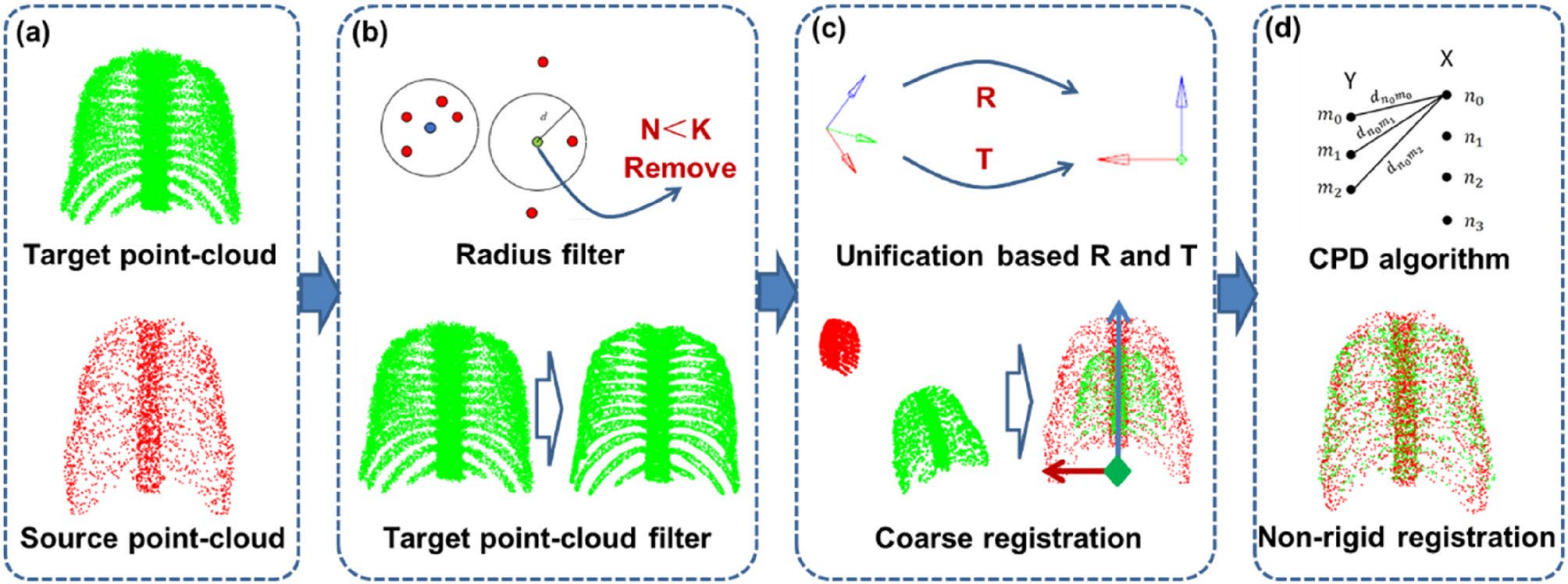
Method overview for generating feature points. (**a**) Point-cloud set; (**b**) Point-cloud filter; (**c**) Coordinate system unification; (**d**) Non-rigid registration.

Figure 3 shows the method overview for automatically generating feature points. Firstly, the target point-cloud extracted from CT data was filtered. Then the coordinate system of target point-cloud and source point-cloud was unified through rough registration. Finally, non-rigid registration was carried out from source point-cloud to target point-cloud to obtain target feature points based on CPD algorithm.

#### Radius filtering of point-clouds

When the point-cloud of target shape was generated from CT data, it usually had a large number of points with outliers (i.e., noise points) in Fig. 3a. These noise points may cause the mismatches during the registration of the target and source point-clouds. Therefore, the radius filter was adopted to remove these noise points. In the area with a point as the center and *d* as the radius, if the number of points is less than *K*, the center point will be removed by the radius filter. In this study, the radius *d* and the number of points *K* was defined as 2 mm and 5, respectively. As shown in Fig. 3b, the noise points in the point-cloud of target shape were significantly reduced by the radius filter. The point-cloud of target shape without noise points was marked as ${P}_{C\_I}$.

#### Coordinate system unification

Usually, there is a huge difference of spatial position between ${P}_{C\_I}$ and ${P}_{S}$, because these two point-clouds were obtained in different coordinate systems. Therefore, it is necessary to unify the coordinate systems of these two point-clouds through matrix operations in Eq. (1).

1 $$ P_{C} = P_{C\_I} *R_{0} + T_{0} $$

where ${P}_{C}$ is the transformed point-cloud of ${P}_{C\_I}$ in the coordinate system of ${P}_{S}$; ${R}_{0}$ is rotation matrix; ${T}_{0}$ is translation matrix.

Using Eq. (1), the difficult step is to find out the rotation and translation matrices. Considering this, the PCA-based coarse registration method was adopted in this study, as shown in Fig. 3c ^25^. In the PCA-based coarse registration, principal component analysis was used to reveal the main distribution direction of point-cloud and reduce dimension of the data. Therefore, the PCA-based coarse registration method is mainly based on the global principal axis direction of point-cloud data for registration. Firstly, the covariance matrix of ${P}_{C\_I}$ and ${P}_{S}$ was calculated. And then, the main feature component, namely the global principal axis direction of the point-cloud data, was calculated according to the covariance matrix to make up the principal axis direction matrices. So, ${R}_{0}$ can be obtained by the principal axis direction matrices by Eq. (2). Finally, the coordinate values of center point calculated from two point-clouds and rotation matrix ${R}_{0}$ were used to obtain the translation matrix ${T}_{0}$ using Eq. (3).

2 $$ R_{0} = U_{P} U_{X}^{ - 1} $$

3 $$ T_{0} = \overline{{P_{S} }} - R_{0} *\overline{{P_{C\_I} }} $$

where ${U}_{X}$ and ${U}_{P}$ are the 3*3 principal axis direction matrices of point-clouds ${P}_{S}$ and ${P}_{C\_I}$, respectively; $\overline{{P }_{S}}$ and $\overline{{P }_{C\_I}}$ are the coordinate values of center point of point-clouds ${P}_{S}$ and ${P}_{C\_I}$, respectively.

#### Non-rigid registration

The ${P}_{C}$ and ${P}_{S}$ were aligned on the principal axis directions after the Coordinate System Unification. However, the mesh morphing technology is based on the same number of source and target feature points to achieve element mapping transformation. Hence it is necessary to create an equal number of source and target feature points in ${P}_{C}$ and ${P}_{S}$. Considering the ${P}_{S}$ as extracted from the outermost nodes of the FEM with high quality mesh, therefore, the ${P}_{S}$ can be directly used as the source feature points. For the target feature points, a non-rigid registration was adapted to mapping the source feature points to the ${P}_{C}$. The target feature points generated by this method not only can be consistent with the source feature points in number, but also have a similar distribution position in human body geometry to the source feature points. This can effectively reduce the distortion of the generating element in the mesh morphing. This non-rigid registration named as alignment depicted in Fig. 3d was realized by the CPD algorithm^26^.

The essence of obtaining target feature points by the non-rigid registration based on the CPD algorithm is to find out an accurate transformation matrix marked as ${\rm T}$. Using this accurate ${\rm T}$ to transform ${P}_{S}$ can obtain a new point set ${P}_{c\_c}$ which deems to be as similar as possible to point-cloud ${P}_{C}$. ${P}_{c\_c}$ can be used as the target feature points in the mesh morphing technology. In order to obtain an accurate ${\rm T}$, the Gaussian Mixture Model (GMM) was adapted to address this problem in CPD algorithm. In the GMM, ${P}_{S}$ was considered as the GMM centroids and ${P}_{C}$ was considered as the GMM generated point-cloud. In other words, ${P}_{S}$ was regarded as a correct standard point-cloud, and ${P}_{C}$ was the point-cloud composed of many scattered points around ${P}_{S}$. The relationship set between ${P}_{S}$ and ${P}_{C}$ in the GMM can be expressed by Eqs. (4) and (5). From Eq. (4), it can be found that the probability of the existence of a point in ${P}_{C}$ was described as the sum of the distance between this point and each GMM centroid (each point in ${P}_{S}$). Since ${P}_{c\_c}$ obtained by transformation of ${P}_{S}$ through the accurate ${\rm T}$ was as completely coincident with ${P}_{C}$ as possible. It should also be pointed out that there is always a point in ${P}_{c\_c}$ that can coincide with the corresponding point in ${P}_{C}$ (the distance between these two points is zero). Therefore, different ${\rm T}$ can be tried repeatedly to transform ${P}_{S}$ to obtain different ${P}_{c\_c}$. The possibility of all point in each ${P}_{c\_c}$ was summed up by Eq. (6). This cumulative sum obtained by Eq. (6) can be used as the rating of the different ${\rm T}$: the greater of the cumulative sum implies the higher score and more accurate of ${\rm T}$. Accordingly, finding the accurate ${\rm T}$ needs to calculate the maximum value of the function described by Eq. (6). By maximizing the likelihood function described in Eq. (6), the parameters ($\theta$ and ${\sigma }^{2}$) in ${\rm T}$ can be obtained. Finally, ${P}_{c\_c}$ were calculated from transformation of ${P}_{S}$ by ${\rm T}$.

4 $$ p\left( {P_{C} } \right) = w\frac{1}{N} + \left( {1 - w} \right)\mathop \sum \limits_{m = 1}^{M} \frac{1}{M}p\left( {\left. {P_{C} } \right|m} \right) $$

where $p\left({P}_{C}\right)$ is represented as the generation probability of each point in ${P}_{C}$; $N$ is the number of points in ${P}_{C}$; $M$ is the number of points in ${P}_{S}$, and $w$ is the weight factor; $p\left(\left.{P}_{C}\right|m\right)$ is the probability of the points in ${P}_{C}$ generated by each point in ${P}_{S}$ and can be calculated by Eq. (5).

5 $$ p\left( {\left. {P_{C} } \right|m} \right) = \frac{1}{{\left( {2\pi \sigma^{2} } \right)^{D/2} }}exp^{{ - \frac{{X - Y_{m}^{2} }}{{2\sigma^{2} }}}} $$

6 $$ E\left( {\theta ,\sigma^{2} } \right) = - \mathop \sum \limits_{n = 1}^{N} log\mathop \sum \limits_{m = 1}^{M + 1} P\left( m \right)p\left( {\left. X \right|m} \right) $$

where $\theta$ is the parameter set consisting of the rotation matrix $R$, translation matrix $T$, and deformation matrix $X$; ${Y}_{m}$ is the center of GMM model; $X$ is the points generated by the GMM model; $D$ is the dimensions of points in ${P}_{C}$ and ${P}_{S}$.

### Mesh morphing technology

After the alignment of ${P}_{C}$ and ${P}_{S}$ in Section "Non-rigid registration", the outermost nodes (point-cloud ${P}_{S}$) in the source FEM were already mapped to the target shape described by ${P}_{C}$. If the internal nodes in the source FEM can be mapped regularly into the target shape as expressed in Eq. (7), implying that target FEM with detailed target geometric characteristics will be obtained. This method for obtaining a new FEM is called as the mesh morphing technology. In 2016, Wang et al.^24^ firstly proposed a mesh morphing technology based on RBF with kernel function TPS (Thin Plate Spline)^27^ expressed as given in Eq. (8). In the RBF with kernel function TPS as given in Eq. (9), the source and target feature points obtained in Section "Automatically generating feature points" was used as the control points to calculate the weight coefficients in Eq. (8) by Eq. (10), then the internal element nodes associated with control points are smoothly transformed from the source FEM to the target FEM by Eq. (7).

7 $$ x_{n}^{\left( T \right)} ,y_{n}^{\left( T \right)} ,z_{n}^{\left( T \right)} = f\left( {x_{n}^{\left( S \right)} ,y_{n}^{\left( S \right)} ,z_{n}^{\left( S \right)} } \right) $$

where $f\left(x,y,z\right)$ is the transformation function for mapping the internal nodes in the source FEM into the geometry of target new model; ${x}_{n}^{\left(S\right)},{y}_{n}^{\left(S\right)},{z}_{n}^{\left(S\right)}$ is the coordinate values of *n* node in the source FEM; ${x}_{n}^{\left(T\right)},{y}_{n}^{\left(T\right)},{z}_{n}^{\left(T\right)}$ is the coordinate values of the corresponding *n* node in the new targe FEM.

8 $$f\left(x,y,z\right)=p\left(x,y,z\right)+{\sum }_{i=1}^{h}{w}_{i}\mathrm{\varnothing }\left(\Vert \left(x,y,z\right)-\left({x}_{i}^{\left({S}^{*}\right)},{y}_{i}^{\left({S}^{*}\right)},{z}_{i}^{\left({S}^{*}\right)}\right)\Vert \right)$$

where $p\left(x,y,z\right)$ is a low order polynomial; $\mathrm{\varnothing }$ is the kernel function representing the TPS in this paper; ${w}_{i}$ is the weight coefficient, ${x}_{i}^{\left({S}^{*}\right)},{y}_{i}^{\left({S}^{*}\right)},{z}_{i}^{\left({S}^{*}\right)}$ is the coordinate values of *i* node in source feature points; $h$ is representing the number of source feature points.

9 $$ \emptyset = \left( {x,y,z} \right) - \left( {x_{i}^{{\left( {S^{*} } \right)}} ,y_{i}^{{\left( {S^{*} } \right)}} ,z_{i}^{{\left( {S^{*} } \right)}} } \right)^{2} log\left( {\left( {x,y,z} \right) - \left( {x_{i}^{{\left( {S^{*} } \right)}} ,y_{i}^{{\left( {S^{*} } \right)}} ,z_{i}^{{\left( {S^{*} } \right)}} } \right)} \right) $$

10 $$ \left[ {\begin{array}{*{20}c} W \\ A \\ \end{array} } \right] = \left[ {\begin{array}{*{20}c} K & Q \\ {Q^{T} } & O \\ \end{array} } \right]^{ - 1} \left[ {\begin{array}{*{20}c} V \\ O \\ \end{array} } \right] $$

where $W=\left[{w}_{1},{w}_{2},{w}_{3},\dots ,{w}_{h}\right]$; $Q$ is represented the coordinate values of the source feature points; $V$ is represented as the coordinate values of the target feature points; $A=\left[{a}_{0},{a}_{x},{a}_{y},{a}_{z}\right]$; $K$ is the distance between each source and its corresponding target feature point.

## Results

In order to verify the method proposed in Section "Method", six skeletal FEMs separated from the adult 50^th^ THUMS including ribcage, pelvis, humerus, radius, tibia, and ulna were successfully morphed into the corresponding target shape extracted from a 3-year-old male child CT data as shown in Table 1. Ribcage, pelvis, humerus, radius, tibia, and ulna FEMs generation required 2.7 s, 1.51 s, 0.932 s, 0.85 s, 0.793 s, and 0.73 s, respectively.

**Table 1:** Summary of source and target FEMs.

Item	Source FEM	Target geometry data	Source and target feature points	Target FEM
ribcage				
pelvis				
humerus				
radius				
tibia				
ulna				

### Geometric error between target shape and FEM

**Figure 4:**
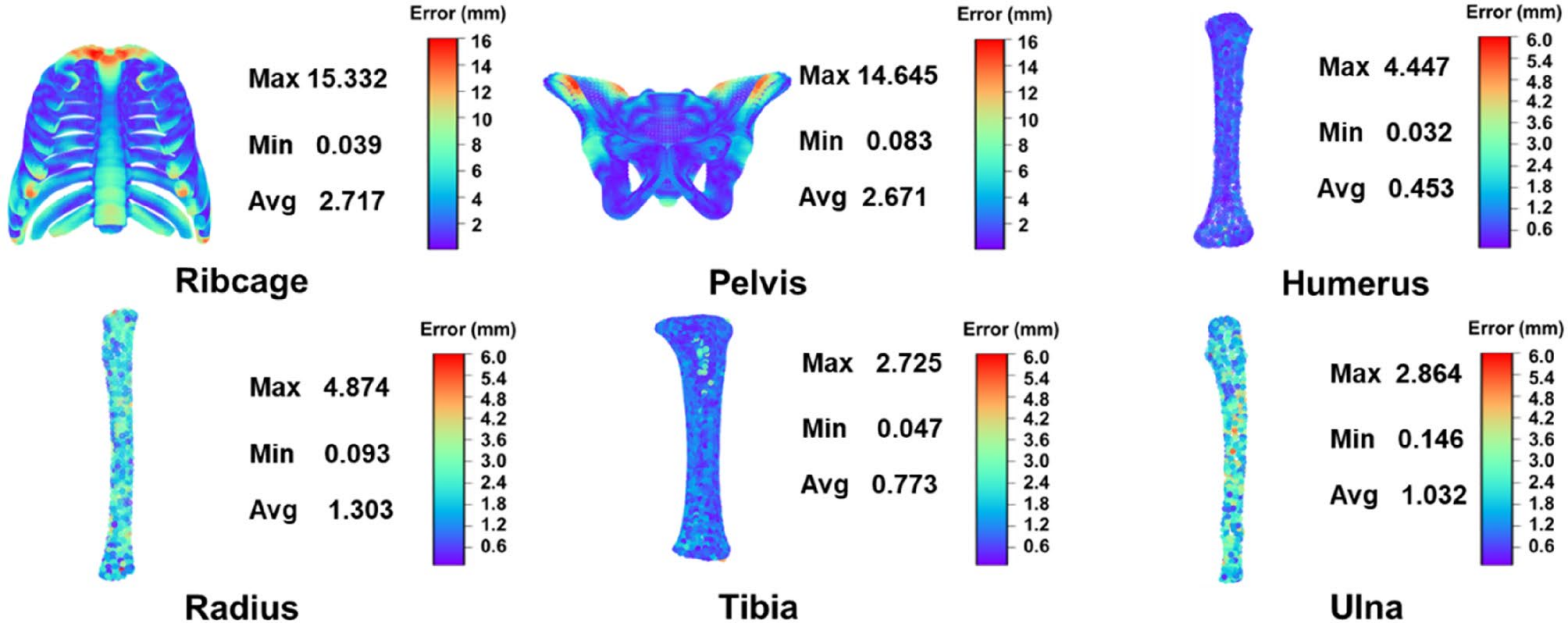
The geometric errors contour of target FEMs.

The distance between the outermost mesh nodes of target FEM and corresponding points of target point-cloud was used to evaluate the geometric error between target shape and FEM as shown in Fig. 4. It can be found that the average geometric error of each model is less than 3 mm, and particularly the average geometric errors of the humerus, radius, tibia and ulna model are less than 1.5 mm. Even the maximum geometric errors of the humerus, radius, tibia and ulna model are less than 5 mm. However, the maximum geometric errors of ribcage and pelvis FEMs are 15.332 and 14.645 mm, respectively. This is mainly due to the filtering of ribcage and pelvis original target point-clouds. The geometric features of ribcage and pelvis are complex, especially their original target point-clouds extracted from CT exhibits a large number of detailed geometric features, thus easily leading to incorrect point-cloud registration. Therefore, their original point-clouds are filtered to reduce useless detailed geometric features. However, the geometric error is the comparison between the target FEM and the original target point-cloud, showing some parts with detailed geometric features are relatively large.

### Mesh quality

The mesh quality of solid elements in target FEMs including Jacobian, Warpage, Skew, Aspect ratio et al. were checked and listed in Table 2, showing that the overall mesh quality of the target FEMs was good and basically meets the requirements of finite element analysis. It was generally considered acceptable if the minimum Jacobian of the mesh is ≥ 0.2. However, it should be noted that the mesh quality of solid elements in target FEMs was a slightly inferior than that of solid elements in source FEMs. For example, compared to the source FEMs, the minimum Jacobians of the target humerus and ulna models were decreased from 0.39 and 0.31 to 0.25 and 0.22, respectively. Because the target feature points generated in Section "Mesh morphing technology" described the detailed geometric features of the target shape as much as possible, this led to a less uniformity of the elements distribution with these target feature points as the outermost nodes and the poor element quality. The mesh quality degraded by this factor can be improved with mesh smoothing.

**Table 2:** Mesh quality of solid elements in target FEMs.

Item	Jacobian		Warpage		Skew		Aspect ratio		Quad faces minimum angel		Quad faces maximum angel	
	≥ 0.5	Minimum	≤ 30	Maximum	≤ 60	Maximum	≤ 8	Maximum	≥ 30	Minimum	≤ 150	Maximum
Target FEMs												
ribcage	99%	0.41	99%	49	99%	57	99%	4	99%	32	99%	153
pelvis	99%	0.9	99%	48	99%	69	99%	4	97%	14	98%	144
humerus	92%	0.25	90%	65	95%	80	94%	15	90%	16	93%	169
radius	96%	0.34	94%	66	96%	70	93%	11	90%	10	90%	159
tibia	90%	0.24	95%	69	95%	86	97%	6	92%	13	92%	170
ulna	82%	0.22	92%	53	97%	71	96%	5	90%	19	88%	185
Source FEMs												
ribcage	99%	0.41	99%	48	99%	57	99%	4	99%	33	99%	154
pelvis	99%	1	99%	48	99%	67	99%	3	98%	15	99%	138
humerus	97%	0.39	95%	49	99%	46	99%	5	99%	33	98%	157
radius	99%	0.39	95%	47	99%	51	99%	5	98%	22	96%	164
tibia	97%	0.32	96%	49	99%	59	99%	5	99%	22	96%	167
ulna	89%	0.31	99%	58	99%	58	99%	5	97%	22	92%	170

## Discussion

In the process of mesh morphing, it was found that the target feature points have significant influences on target FEMs. In this section, the influences of the generation methods and number of target feature points on the geometric error and mesh quality of target FEMs will be discussed.

### The influences of generation method of feature points

In this study, the non-rigid registration based on the CPD algorithm was adapted in Section "Automatically generating feature points" to obtain the target feature points. In the non-rigid registration, using coordinate translation, rotation, scaling and local deformation to generate target feature points from source feature points can better describe the geometric characteristics of the target point-cloud. In fact, the target feature points can also be obtained by rigid registration. For CPD algorithm, rigid registration is a transformation that does not change the relative position between two points in the point-cloud, including translation, rotation and scaling. Non-rigid registration can change the relative position between two points in the point-cloud. It can cause local deformation by nonlinear matrix. We did both registrations by changing the $\theta$ in Eq. (6). The $\theta$ in rigid registration include rotation matrix $R$, translation matrix $T$ and scaling matrix $S$. Instead of rigid registration, the $\theta$ contains an additional nonlinear matrix $N$.

**Figure 5:**
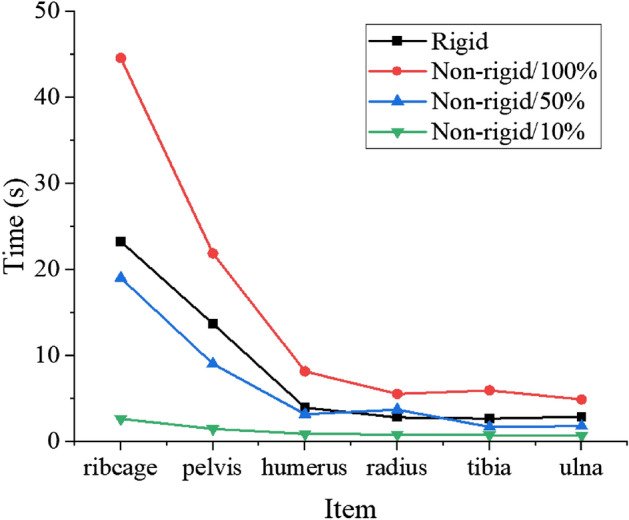
Comparison of the generation time of different bone FEMs under different generation methods and number of feature points.

As shown in Fig. 5, when using non-rigid registration, ribcage, pelvis, humerus, radius, tibia, and ulna FEMs generation required 44.61 s, 21.9 s, 8.192 s, 5.581 s, 5.988 s, and 4.933 s respectively. However, due to the rigid registration ignores the nonlinear transformation, the FEMs generation time reduced to 23.285 s, 13.724 s, 3.972 s, 2.846 s, 2.714 s, 2.911 s respectively.

**Figure 6:**
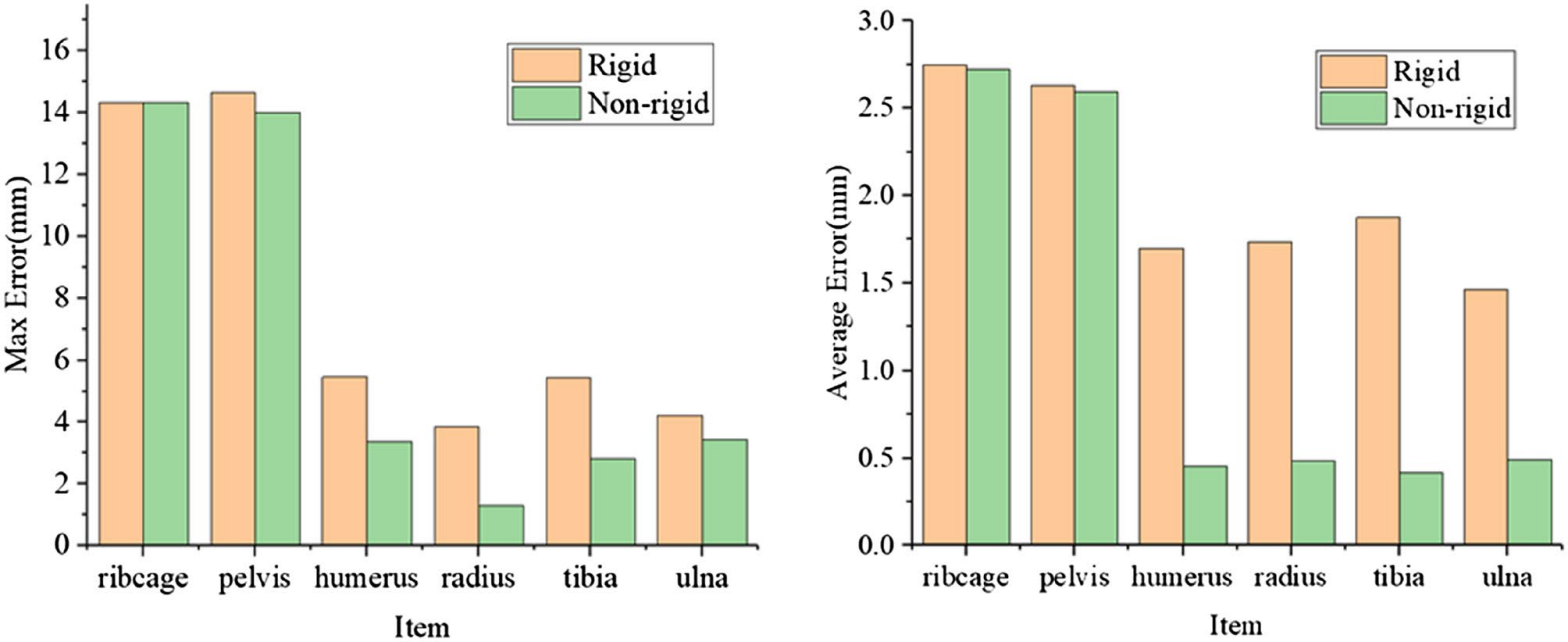
The comparison of geometric errors of target FEMs generated by rigid and non-rigid registration.

In Fig. 6, the geometric errors including maximum and average errors of target FEMs generated by the non-rigid registration method are lower than that generated by the rigid registration. This is mainly because the nonlinear transformation is not adapted to make the target feature points closer to the geometric features of the target shape in the rigid registration method. Especially for humerus, radius, tibia, and ulna, their detailed geometric characteristics of source and target point-clouds differ greatly. Therefore, compared with target FEMs generated by the rigid registration, the average geometric error of target FEMs generated by the non-rigid registration are decreased by 73.3%, 72.2%, 77.7% and 66.5%, respectively.

**Figure 7:**
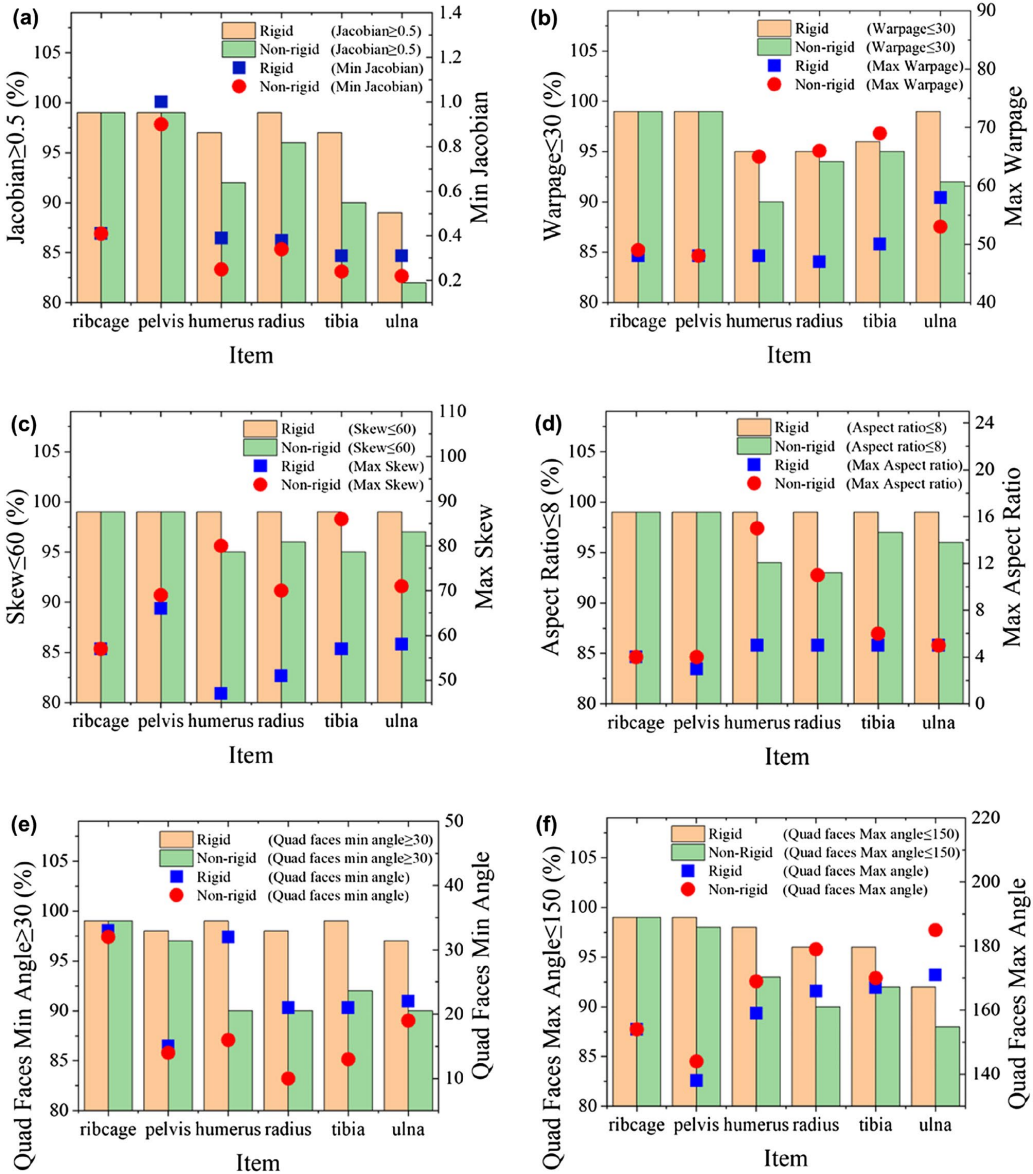
Comparisons of mesh quality of the corresponding target FEMs generated by rigid and non-rigid registration method. (**a**) Jacobian; (**b**) Warpage; (**c**) Skew; (**d**) Aspect Ratio; (**e**) Quad faces Min Angle; (**f**) Quad faces Max Angle.

Figure 7 shows that the mesh quality of target FEMs generated by rigid registration are similar to that of source FEMs shown in Table 2. This is mainly because the translation, rotation and scaling in the rigid registration method did not cause local deformation of the mesh elements to affect the mesh quality of target FEMs. Therefore, the mesh quality of target FEMs generated by rigid registration were also higher than those of the corresponding target FEMs generated by non-rigid registration.

### The influences of number of target feature points

Different number of target feature points have different functional capability to describe the geometric features of target point-cloud, affecting the generation of nodes for the target FEMs in the mesh morphing process. Therefore, the influences of number of target feature points were described in this section. Selecting 100%, 50%, and 10% of source features points by the uniform sampling method were respectively used to generate the corresponding number of target feature points by non-rigid registration for further generating target FEMs. It can be seen from Fig. 5, as the number of feature points decreases, the generation time of target FEMs decreases gradually. When 10% of source features points are selected, the ribcage FEM with 27,728 elements needs only 2.7 s.

**Figure 8:**
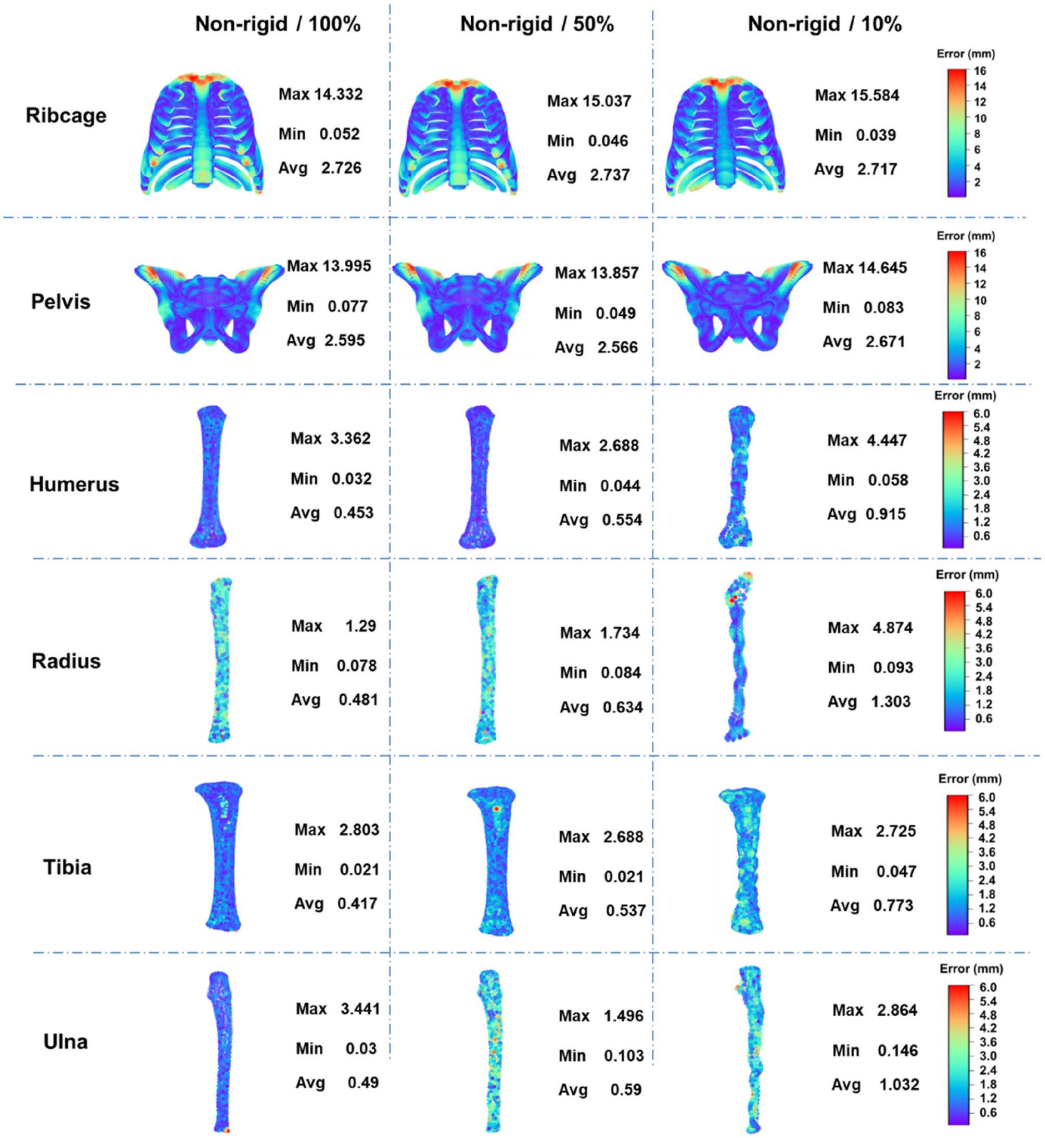
Geometric errors of target finite element model generated with different number of feature points.

Figure 8 shows the geometric errors including the maximum and average errors of target FEMs increased with decreasing of the number of target feature points. The influences of number of target feature points on the geometric errors are slightly in the target FEMs of ribcage and pelvis, but significantly in the target FEMs of humerus, radius, tibia, and ulna. This is primarily due to the detailed geometric characteristics of their source and target point-clouds differ significantly. Therefore, more target feature points are necessary to describe the detailed geometric characteristics of target point-clouds.

Data shown in Table 3, reveal the mesh quality of the target FEMs generated by the small number of target feature points is better. This is mainly due to fewer target feature points that ignore some detailed geometric features of the garget point-cloud to reduce the local deformation of mesh elements. Similarly, in the general mesh generation, it is also important and necessary to balance the mesh quality and the details of geometric features.

**Table 3:** Mesh quality of corresponding target FEMs generated by different number of target feature points.

Item	Jacobian		Warpage		Skew			Aspect ratio			Quad faces minimum angel			Quad faces maximum angel	
	≥ 0.5	Minimum	≤ 30	Maximum	≤ 60	Maximum	≤ 8		Maximum	≥ 30		Minimum	≤ 150		Maximum
10%															
ribcage	99%	0.41	99%	49	99%	57	99%		4	99%		32	99%		153
pelvis	99%	0.8	99%	48	99%	69	99%		4	97%		14	98%		144
humerus	92%	0.25	90%	65	95%	80	94%		15	90%		16	93%		169
radius	96%	0.34	94%	66	96%	70	93%		11	90%		10	90%		159
tibia	90%	0.24	95%	69	95%	86	97%		6	92%		13	92%		170
ulna	82%	0.22	92%	53	97%	71	96%		5	90%		19	88%		185
50%															
ribcage	99%	0.41	99%	50	99%	57	99%		4	99%		32	99%		153
pelvis	99%	0.9	99%	48	99%	70	99%		4	98%		13	98%		145
humerus	86%	0.14	86%	86	95%	77	90%		18	91%		13	88%		198
radius	89%	0.14	80%	93	95%	78	91%		17	91%		15	90%		194
tibia	90%	0.13	84%	98	97%	85	97%		17	89%		14	89%		194
ulna	88%	0.18	86%	99	97%	75	95%		16	92%		6	85%		187
100%															
ribcage	99%	0.40	99%	50	99%	57	99%		5	99%		32	99%		153
pelvis	99%	0.8	99%	48	99%	68	99%		3	99%		14	98%		139
humerus	85%	0.12	80%	96	95%	84	87%		25	90%		11	87%		253
radius	89%	0.08	82%	103	98%	73	87%		20	90%		16	90%		231
tibia	90%	0.05	80%	98	97%	82	97%		25	94%		14	91%		256
ulna	87%	0.08	81%	99	97%	80	93%		18	90%		8	84%		254

### Limitations

Through presentation and discussion of the method proposed in this study, the source FEMs can be quickly morphed to the target FEMs more consistent with the geometric features of the target point-cloud. However, in order to describe more detailed geometric feature of target point-cloud, a non-rigid registration method is used to locally deform the source feature points to generate target feature points. This local deformation caused a decrease in the mesh quality of the target FEM compared to that of the corresponding source FEM. Especially when the detailed geometric features of the source and target point-clouds differ significantly, the mesh quality of the generated target FEMs decreases more significantly. For the meshes with acceptable quality, we usually improve them by smoothing, elements optimization, nodes optimization and so on. Therefore, it is necessary to conduct more in-depth study on how to balance the mesh quality and the details of geometric features of the target point-cloud. In addition, only the human skeletal FEMs were considered in this study, the whole human body FEM also needs to be investigated in future research studies.

## Conclusion

In this study, a fast-morphing methodology for generating human skeletal FEM with detailed geometric features based on CPD and RBF algorithms was proposed. This is indeed to be the first time that the CPD algorithm is applied to the traditional mesh morphing technology. Using this algorithm enables realization of the improvement from manually placing feature points to automatically generating feature points. In addition, this method can directly use the point-cloud of the target geometric data for finite element modeling to save a lot of work on the reverse modeling. This fast-morphing methodology was successfully used to morph several human skeletal FEMs extracted from THUMS adult model to 3-year-old child skeletal FEMs. The morphing results proved that the human skeletal FEMs generated by this fast-morphing methodology has resulted in small geometric errors and high mesh quality. Limitations on this approach will be continued to be investigated further in research pertaining to this area in the future.

## Data Availability

The datasets used and analyzed during the current study available from the corresponding author on reasonable request.
